# Atypical Eclampsia in a Pregnant Woman Infected by COVID-19

**DOI:** 10.1155/2022/9952355

**Published:** 2022-04-27

**Authors:** Maha Ahmad Odeh, Yousef S. Abuzneid, Omar Badareen, Khaled Masarweh

**Affiliations:** ^1^Al-Quds University Faculty of Medicine, Jerusalem, State of Palestine; ^2^Makassed Islamic Charitable Society, Jerusalem, State of Palestine

## Abstract

The coronavirus disease 2019, also called (COVID-19), is an infectious disease which is caused by a virus called severe acute respiratory syndrome coronavirus 2 (SARS-CoV-2). The first report was in December 2019, and on March 12, 2020, the World Health Organization (WHO) declared this disease a pandemic. COVID-19 targets many major organs causing life-threatening systemic complications. It can cause lung damage and respiratory failure in addition to systemic inflammation and immune dysregulation. Hypercoagulable state and numerous neurological abnormalities also have been reported due to this condition. Going through the literature review, we found some cases of pregnant women with novel coronavirus infection, being mostly mild illnesses, and most of these cases were focused on maternal-fetal transmission and neonatal outcomes. In this case report, we present the case of a COVID-19 positive woman who came to our emergency department at 34 weeks of gestation with tonic-clonic seizures. This case was a challenge for us because we faced a new an unknown manifestation of both COVID and eclampsia.

## 1. Introduction

An increased risk for perinatal complications due to COVID-19 in pregnant patients has been recently reported, including higher incidence of preeclampsia and preterm birth. Even if the mechanisms are currently unknown, some theories explain that SARS-CoV-2 affects multiple cell types in the human placenta by colonization. One of the mechanisms is associated with renin-angiotensin hormonal system (RAS) alterations in the placenta, which lead to increase blood pressure and preeclampsia. These studies in pregnant women support a link between the virus and the development of preeclampsia [1, 2].

During pregnancy, the most common medical disorder is hypertension. Hence, gestational hypertension-preeclampsia is used to describe a huge spectrum of disorders for those patients who may have just mild elevation in blood pressure, severe hypertension with variable involvement of different organs and hemolysis, elevated liver enzymes, and low platelets (HELLP) syndrome [3].

During pregnancy, the new onset of convulsions and/or coma or in the immediate postpartum period and in the presence of preeclampsia is defined as eclampsia. Its cause is unknown, and it occurs in 0.3%-0.6% globally. Mental status, frontal and/or occipital headaches, nausea, and/or visual changes are usually symptoms that precede these convulsions or seizures [4, 5].

According to the literature review, more than 35% of the patients that suffer from COVID-19 develop neurological symptoms; however, the prevalence of these signs and symptoms is higher in patients with severe COVID-19 infection, being cerebral hypoxia after a COVID-19 respiratory failure one of the possible causes [6].

From these symptoms, headache is the most common one, with a prevalence varying from 6.5 to 23% and a mean prevalence of 8% [7].

The elderly and patients with preexisting chronic medical diseases are at a higher risk of neurological squeal of acute infections (e.g., impaired consciousness or delirium). These patients may also experience encephalopathy and confusion [8].

Many articles had been published about COVID-19 cases suffering from recurrent transient generalized tonic-clonic seizures. However, neither or those cases had a history of epileptic seizures nor a family history of seizure disorders. Different explanations have been theorized to explain COVID-19–associated epilepsy. Some explain it as a neuronal hyperexcitability because of increase secretions of inflammatory cytokines, tumor necrotizing *α*, and the granulocyte colony-stimulating factor, through activation of glutamate receptors leading to episodic seizures.

In some other papers, it has been mentioned that the seizures may be caused due to direct COVID-19 encephalitis and invasion to the brain. However, there is no stipulated evidence whether the SARS-COV-2 virus can or cannot cross the blood-brain barrier (BBB) [9, 10].

## 2. Case Presentation

A 28-year-old pregnant woman with an estimated gestational age of 34 weeks +3 days and COVID positive, presented to the emergency department after two attacks of tonic-clonic seizure (with tongue biting, up rolling eyes) which lasted one minute each and were associated with loss of consciousness. The patient was given 10 mg diazepam IV as a first line treatment in the ER by our doctors.

She was confirmed to be COVID-19 positive by a polymerase chain reaction (PCR) by throat swab four days before the tonic-clonic seizures occurred. The seizures were not associated with nausea or vomiting, headache, abdominal pain, vision disturbances, shortness of breath, chest pain, nor lower limb edema. There were no reports of proteinuria or hypertension in her antenatal care.

The patient had no history of eclampsia or preeclampsia in her previous pregnancies and no history of epilepsy or trauma to the abdomen or abdominal manipulations prior to the onset of symptoms.

The patient's current pregnancy was uneventful, with no known passage of show, drainage of liquor amnii, vaginal bleeding, or other genitourinary symptoms, and fetal movements were regular. The pregnancy was booked for antenatal care, and she had detailed ultrasound scans that were normal and confirmatory of a healthy gestation. There were no remarkable gynecologic symptoms or evidence of obstetric complications. Physical examination was also unremarkable.

The patient's vital signs were stable with a heart rate of 95 beats/min, blood pressure of 110/65 mmHg in supine position, a temperature of 36.2 °C, oxygen saturation of 99%, and respiratory rate of 18 cycles/min.

The abdomen was enlarged and moving with respiration as expected. There was a Pfannenstiel scar due to previous CS, but there was no abdominal tenderness. The symphysio-fundal height was consistent with an intrauterine pregnancy of 34 weeks and compatible with her gestational age by date, being the fetal lie longitudinal with cephalic presentation.

No contractions were noticed, and regular fetal heart tone was heard. The vulva and vagina were normal. The cervix was not effaced, and the cervical os was closed. Lower limbs were symmetrical with no edema noted and deep tendon reflexes +2.

On a bedside transabdominal ultrasound, a single viable fetus was seen, cephalic, with normal liquor amnii depth of 5 cm and a placenta that was normally positioned.

After a detailed history and physical examination were taken and the confirmation of a viable pregnancy with no complications was done, baseline investigations were performed. The patient's CBC showed a hemoglobin level of 9.1 (normal range is 12.0-16.0 g/dL), MCV of 71.1 (normal range is 80-97 fL), and high RDW (16.7% when the normal range is 11.6-14.8%). White blood cells were slightly elevated (11.3 K/*μ*L when the normal range is 4.690-10.2 K/*μ*L). Neutrophils percentage was 76%, lymphocytes percentage was 18%, and PLTs count was 280 K/*μ*L. The patient's blood work also showed a CRP of 85.9 (normal range is 0-6 mg/L), BUN of 5.2 (normal range is 6-20 mg/dL), and very high LDH (334 when the normal range is 135-225 U/L), and albumin and calcium were both low (albumin 2.2 when the normal range is 3.8-4.4 g/L and calcium 7.57 when the normal range is 8.6-10.2 mg/dl). Liver function for total protein was low 5 g/dl (normal range is 6.6-8.7 g/dl), and the kidney function test and coagulation profile were normal. The urine analysis showed trace proteins 22.87 mg/dl (normal range 0-15 mg/dl).

The patient's vital signs were stable, and all her blood pressure readings were less than 120/80 mmHg.

Administration of 5 g of magnesium sulfate was started as a loading dose over 15 minutes and then 1 g/hr with close monitoring. This was followed by two doses of dexamethasone and an urgent C-section, with no complications during and postoperative surgery.

**Figure 1:**
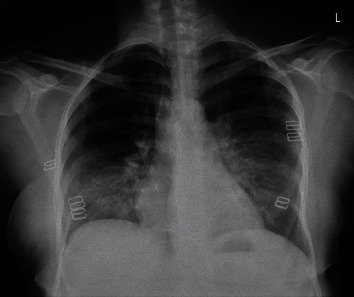
Brain CT scan showing no space occupying lesions nor hemorrhage in the parenchyma, a normal ventricular system with no hydrocephalus and no midline shift.

**Figure 2:**
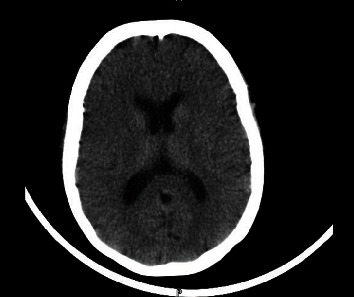
Chest X-ray showing small consolidations in both lower lobes with linear atelectatic changes.

The next day, a brain CT scan was performed showing no space occupying lesions nor hemorrhage in the parenchyma, a normal ventricular system with no hydrocephalus and no midline shift. Posterior fossa elements and cerebellopontine angles were within normal limits, sinuses were well and clear, and there were no significant focal bony lesions (Figure 1). A chest X-ray was done and showed small consolidations in both lower lobes with linear atelectatic changes (Figure 2).

The patient's symptoms were dramatically improved after receiving Diazepam in the emergency department, in addition to magnesium sulfate and with the termination of her pregnancy. She had a satisfactory postoperative recovery and was discharged after 4 days against medical advice and with a recommendation to do further follow-ups.

## 3. Discussion

Preeclampsia was previously considered a triad of hypertension, proteinuria, and edema. However, edema incidence without any complication during pregnancy made it no longer considered an important part of preeclampsia. Nowadays, there is also a term to describe the atypical hypertensive disorders during pregnancy, which is “atypical preeclampsia-eclampsia” [11].

The definition of atypical preeclampsia-eclampsia is still unclear; however, the literature includes cases with hypertension but no or minimal proteinuria, marginally elevated hypertension with proteinuria or without neither proteinuria nor hypertension [11]. This condition commonly manifest postpartum after 48 hours or more or before 20 gestational weeks, in patients resistant to MgSO_4_ treatment, in addition to those who have HELLP syndrome.

It has been thought that SARS-CoV-2 uses an angiotensin-converting enzyme as an entry receptor to the cells via a protein called viral spike (S) that binds the N-terminal peptidase domain of ACE2 (which recent studies confirmed that it is expressed in placental cells). This enzyme is a key component of the renin-angiotensin system (RAS) that controls blood pressure, angiogenesis, and fetal development by regulating inflammation [12, 13]. Some studies demonstrate that the virus spread to stromal cells, fetal trophoblasts, and macrophages in the placenta, cells that express the ACE2 receptor. This will lead to a decrease in those receptors and will result in an alteration of the RAS pathway (changes that are noted in preeclampsia) [13, 14].

We should also mention the significant role in the balance between angiogenic (e.g., PlGF) and antiangiogenic (e.g., sFlt1) factors that are required for normal placental angiogenesis. SARS-COV-2 causes dysregulation of PlGF and sFlt1 in infected placentas, suggesting a mechanism for preeclampsia. The virus causes multiorgan failure by endothelial dysfunction which is caused by an increase in the expression of antiangiogenic factors and downregulation of the angiogenic factors.

Some clinical studies are showing a strong link between SARS-CoV-2 infection and preeclampsia probability by comparing preeclampsia in severe infected patients versus mild to moderate infected pregnant groups [14, 15].

Coagulopathy and fibrin deposition in the placenta of infected women are also listed as a possible explanation for preeclampsia due to SARS-CoV-2 [14].

As mentioned before, COVID-19 could cause a multi-organic failure. The main manifestation of COVID-19 is flu-like symptoms in addition to ageusia and anosmia.

Previous research on other coronaviruses such as Middle East Respiratory Syndrome (MERS) and SARS were documented to be associated with an increased in the incidence of preeclampsia [16].

This virus has various manifestations, as we mentioned earlier in this paper, but in this case report, we focus on the nervous system by the involvement of the central and peripheral systems, causing symptoms that vary from mild (headache, dizziness, taste, and smell impairment) to severe (acute cerebrovascular diseases, encephalopathy, ataxia, skeletal muscle manifestations, viral encephalitis, seizures, and death) [6, 17].

As mentioned previously, data on atypical eclampsia and COVID-19 infection in pregnancy is limited. In this case, atypical preeclampsia-eclampsia was suspected (even though the patient had trace proteinuria with normal blood pressure readings) because she had two attacks of tonic-clonic seizures [18].

Classically, the disease affects the arteries and kidneys, where it will manifest as proteinuria and hypertension; furthermore, many organs will also be affected. In atypical cases, however, it manifests as cerebral involvement that presents as eclampsia [11]. While treating the patient presented in this case study, we encountered two possibilities: We either consider the tonic-clonic seizures a manifestation of eclampsia and terminate the pregnancy or consider them a complication of COVID-19 and treat her supportively.

## 4. Conclusion

The role of SARS-CoV-2 in the placenta is still poorly understood and we need further investigations about it, including the transmission mechanisms, fetal infection, and its consequences.

The diagnosis of preeclampsia/eclampsia should be suspected even the absence of hypertension or proteinuria. It is important to widen the spectrum of the definition of preeclampsia-eclampsia since there are cases in which there is hypertension without proteinuria and vice versa. Moreover, one should be careful to avoid misdiagnosis of other preexisting conditions, such as undiagnosed chronic hypertension or renal disease that might lead to unnecessary interventions.

Therefore, a detailed history is crucial to assess for the presence of symptoms and to obtain targeted laboratory tests to confirm the diagnosis of atypical preeclampsia.

This case is a demonstration of the uncertainty about this subject and the need for more research in this area. Hence, careful monitoring of symptoms may help prevent morbidity and mortality.

## Data Availability

The data supporting this case report are from previously reported studies and datasets, which have been cited.
